# Deep Learning Analysis of Polish Electronic Health Records for Diagnosis Prediction in Patients with Cardiovascular Diseases

**DOI:** 10.3390/jpm12060869

**Published:** 2022-05-25

**Authors:** Kristof Anetta, Ales Horak, Wojciech Wojakowski, Krystian Wita, Tomasz Jadczyk

**Affiliations:** 1 Natural Language Processing Centre, Faculty of Informatics, Masaryk University, 602 00 Brno, Czech Republic; xanetta@fi.muni.cz; 2 Department of Cardiology and Structural Heart Diseases, School of Medicine in Katowice, Medical University of Silesia, 40-055 Katowice, Poland; wojtek.wojakowski@gmail.com; 3 First Department of Cardiology, Medical University of Silesia, 40-055 Katowice, Poland; welwetek@poczta.onet.pl; 4 Interventional Cardiac Electrophysiology Group, International Clinical Research Center, St. Anne’s University Hospital Brno, 656 91 Brno, Czech Republic

**Keywords:** electronic health records, deep learning, text analysis, diagnosis prediction, Polish language

## Abstract

Electronic health records naturally contain most of the medical information in the form of doctor’s notes as unstructured or semi-structured texts. Current deep learning text analysis approaches allow researchers to reveal the inner semantics of text information and even identify hidden consequences that can offer extra decision support to doctors. In the presented article, we offer a new automated analysis of Polish summary texts of patient hospitalizations. The presented models were found to be able to predict the final diagnosis with almost 70% accuracy based just on the patient’s medical history (only 132 words on average), with possible accuracy increases when adding further sentences from hospitalization results; even one sentence was found to improve the results by 4%, and the best accuracy of 78% was achieved with five extra sentences. In addition to detailed descriptions of the data and methodology, we present an evaluation of the analysis using more than 50,000 Polish cardiology patient texts and dive into a detailed error analysis of the approach. The results indicate that the deep analysis of just the medical history summary can suggest the direction of diagnosis with a high probability that can be further increased just by supplementing the records with further examination results.

## 1. Introduction

Cardiovascular diseases (CVDs) are the leading cause of mortality and morbidity worldwide, and ischemic heart disease (IHD), also known as coronary artery diseases (CADs), represents one of the major global health burdens [1]. Subsequently, a large number of CVD patients treated in outpatient and inpatient settings generates extensive amounts of medical data, and current clinical practice requires the documentation of every medical encounter. Routinely, medical professionals collect information related to patients that result in textual documents (admission notes, medical histories, physical examinations, progress notes, diagnostic and laboratory test results, discharge summaries and recommendations) that are stored in an electronic health record (EHR) system with the final coding-based classification of the diseases and procedures. To facilitate categorization, the International Statistical Classification of Diseases and Related Health Problems (ICD), now one of the most widely used systems among healthcare systems worldwide, was introduced to provide information on causes, severity and consequences of diseases. Primarily, the ICD codifies diagnoses of diseases into alphanumeric codes for public health, statistical and reimbursement purposes [2]. However, despite advancements in information technologies and EHR systems, ICD coding is still based on a manual and time-consuming approach (~30 min per case by disease coders) [3], causing even more workload for medical professionals who spend almost half of their work time on electronic documentation [4]. Thus, automated ICD coding systems have the potential to support providers in everyday clinical practice and help medical organizations to optimize workflows. Furthermore, correctness of ICD coding has a direct impact on the billing process. In general, health insurance systems (e.g., Medicare) reimburse hospitals in a fee-for-service model based on reported ICD codes [5]. Thus, misclassification might result in inappropriate reimbursement for medical organizations. Accordingly, the accurate categorization of diagnoses and provided treatment has a realistic influence on clinical settings from a broad perspective.

EHRs consist of possibly large volumes of heterogeneous data that include databases of form-like information based on both external and internal standards, textual narratives by doctors and medical specialists, and imaging data from patients’ investigations. Automated analyses of EHR data may lead to fast and accurate predictions of many aspects of the treatment such as patient diagnoses or risk prediction [6,7], disease progression [8], and unplanned readmission to hospitals [9]. The topic of diagnosis prediction is usually based on data such as the temporal sequence of patient’s visits represented as medical codes of the investigations. Ma et al. [10] experimented with deep learning architecture based on bidirectional recurrent neural networks supplemented with attention mechanisms, which allowed them to improve the processing of long medical code sequences to reach a prediction accuracy of 46–48%. Gao et al. [11] used co-attention memory networks that allowed them to combine the sequential history data with patient demographic information using specific diagnosis embeddings trained over a disease taxonomy, ultimately achieving a mean average precision MAP@5 of 57%.

The textual parts of EHRs together form 80% of patients’ information without any straightforward technique of extracting the information for subsequent applications [12]. However, current natural language processing (NLP) approaches based on advanced deep learning techniques open new ways of prospering from the full EHR content [13]. The best deep language models are based on encoder–decoder transformer architectures [14], which are pre-trained on very large collections of unstructured texts such as BERT [15] and RoBERTa [16]. The final analysis accuracy of these models directly corresponds to the input texts and tasks used for pre-training, as well as the language(s) of the training texts and the internal size of the architecture model; the numbers of trainable parameters range from 110 million in BERT-base to 354 million in RoBERTa-large. Other transformer language models with even larger numbers of parameters exist, e.g., GPT-3 [17], but they are usually pre-trained and designed for text generation tasks.

In the presented study, we employed the latest large transformer models for the Polish language, which was used as a representative of non-mainstream languages. The deep semantic representation of EHR input texts was then trained and evaluated in the task of predicting the final patient diagnosis ICD code based on a selected textual part of his or her EHR record. According to a mix of patient medical history and selected information from further examinations, the prediction accuracy was found to range from 69% to 78% when distinguishing between the four most frequent cardiological diagnoses that cover 75% of hospitalizations, and the accuracy was also shown to significantly surpass several baseline approaches even with increasing numbers of diagnoses.

## 2. Materials and Methods

Before discussing any details of the dataset, it is necessary to emphasize the aspects of language and data availability: while English boasts a booming field of deep learning applications in EHR analysis and massive health record databases have been made public [18,19], the situation is radically different in other languages, especially in non-mainstream languages such as Polish. Due to the legal difficulties of obtaining health record data, this field of research remains largely untouched in many languages, and for this reason, tens of millions of people are unable to benefit from AI-enhanced medicine.

It is hard to overstate the importance of the fact that this experiment was conducted with Polish health records—as such, it is a pioneering endeavor leading the way for neighboring Slavic languages (such as Czech and Slovak) and many others facing similar data and resource scarcity.

### 2.1. Dataset Characteristics

The dataset for this study was extracted from the Asseco Medical Management Solutions (Asseco Poland S.A., Rzeszow, Poland) EHR system covering the patient population hospitalized at the 3rd Department of Cardiology, Leszek Giec Upper Silesian Medical Centre of the Medical University of Silesia in Katowice (GCM), Poland.

The study adhered to the principles of the Declaration of Helsinki and the Good Clinical Practice guideline. Prior to analysis, patient data were anonymized, and a data privacy policy was applied in accordance with the General Data Protection Regulation (GDPR) [20].

The dataset consisted of 50,465 recorded cardiology hospitalizations between 2003 and 2020 (see more dataset statistics in Table 1). As can be seen in the example in Table 2, each record contains up to four sections of unstructured text and one piece of structured data: the ICD-10 code of the final diagnosis. The record always contains only one ICD-10 diagnosis code—even though this often does not correspond to real-world states of multiple diagnoses that are simultaneously present, it allows for a straightforward classification task that tries to identify the most salient diagnosis.

The four textual parts of each record correspond to 4 sections along the timeline of the hospitalization:

1. Admission, reasons for admission, and medical history.
2. Physical examination at admission.
3. Discharge, summary of hospitalization, and results.
4. Recommendations at discharge and medication.

For the purposes of content analysis and exploitation, the individual sections have a varying degree of utility. Section 2 contains template-based records of physical examination, which differ very little from each other and bear no strong relation to the diagnosis (the nuances of cardiac function rarely have specific outward manifestations). Section 4 mostly contains lists of medication prescriptions and therefore lacks both standard words and natural language syntax, which makes it unsuitable for the natural language-based deep learning methods utilized in our experiments. On the other hand, Sections 1 and 3 contain assessments custom-written by doctors before and after the hospitalization, and these provide the highest-quality language data in the dataset. Therefore, our focus was primarily directed at:

- Section 1 (admission) composed without the knowledge of future diagnosis; models trained on Section 1 text can be said to perform true prediction.
- Section 3 (discharge) composed with the knowledge of the diagnosis; models trained on Section 3 text are useful for pattern/inconsistency discovery but also set a ceiling value for any prediction efforts, revealing the limitedness of textual information (as distinct from measurements or medical imaging) in determining the actual physical condition of a patient.

### 2.2. Dataset Preprocessing for Classification

The described dataset was subsequently used to run a deep learning classification experiment aimed at predicting the ICD-10 diagnosis category based on the unstructured text of the health records. For this experiment, we used state-of-the-art transformer language models, RoBERTa and BERT, trained on Polish or multilingual data, and we fine-tuned them for multi-label text classification. The training data used for fine-tuning consisted of selected parts of the unstructured health record text, labeled with the respective final ICD-10 diagnosis category.

Since BERT and RoBERTa can only be fine-tuned with sequences of up to 512 tokens (words and punctuation), the training data were appropriately resized, even though no shortening was necessary in most cases thanks to the health records being already felicitously suited to this limitation.

While the full ICD-10 codes available in the dataset have 4 characters (e.g., I25.0), we decided to only use the first 3 characters marking the diagnosis category (I25.0, I25.1, and I25.2 collectively as I25). Apart from providing a more appropriate starting granularity, this setup benefits from larger class sizes.

### 2.3. Limitations and Data Consistency Considerations

The presented approach is concentrated on identifying the core information in a representative collection of summary documents per each final diagnosis. In this respect, the prediction accuracy depends on the available number of documents per category.

**Figure 1:**
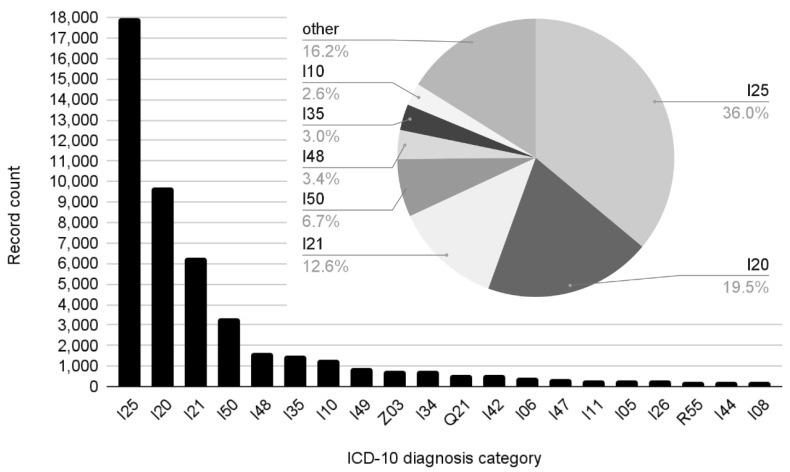
Representation of top 20 concluding ICD-10 categories in the data (bar chart) and proportions of categories with more than 1000 examples (pie chart).

Even with 3-character ICD-10 categories, the problem faced by the classification task was the high number of classes (170) in the dataset and the stark differences in their frequency (see Figure 1 and Table 3), where the leading four categories (I25, I20, I21, and I50) accounted for ¾ of the total (74.85%) and the bottom 150 comprised a mere 4.04%, with 13 examples per category on average; the long tail of this decline curve was unsuitable for deep learning.

To deal with these imbalances and data scarcity in the underrepresented categories, we created different subsets of training data using a limited number of the most frequent categories, subsuming the tail of the data under the “other” category. Table 4 shows the key training subsets.

In the descriptions of admission and discharge notes, doctors focus on the main medical problem that patients present during hospitalization. As the whole studied database came from a hospital focused on cardiovascular diseases, most of the non-cardiological chronic conditions will not be mentioned in this paper unless the disease was shown to have a direct impact on the current cardiovascular problem (e.g., hyperthyroidism and atrial fibrillation or chest pain and low hemoglobin level).

Having been collected over 18 years, the data exhibit natural variations in diagnosing practices, which may reflect a variety of influences, including:

- Real changes in disease prevalence.
- Evolution of medical research.
- Individual staff members’ documentation tendencies.

**Figure 2:**
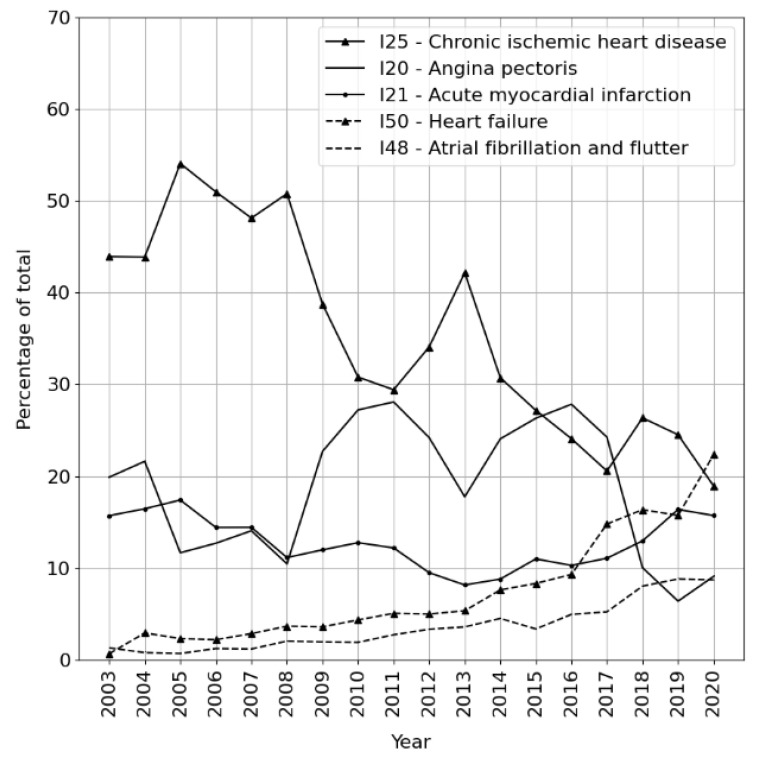
Development of the proportions of 5 most frequent primary diagnoses in the dataset between 2003 and 2020.

For example, Figure 2 shows that since 2003, I25 (chronic IHD) has been declining in favor of the more specific ICD-10 categories. This aspect of the dataset might be the subject of future experiments considering features of health records in relation to the year of diagnosis.

## 3. Results

### Experiments

The state-of-the-art models chosen for fine-tuning in the classification task (their performance in general Polish measured by the KLEJ benchmark [21]) included HerBERT [22], Polish RoBERTa [23], and the multilingual XLM-RoBERTa [24]. The best-performing model in most cases was Polish RoBERTa, even though HerBERT sometimes prevailed in cases with higher numbers of categories. In further analysis results, Polish RoBERTa was used as our chosen model.

In preparing the training subsets, we divided the entire dataset into files based on both the section of the report and its final three-character ICD-10 category (one file per category per section, e.g., “all sections 1 belonging to I25”). For each subset, we created a different “other” data file containing a random selection of the remaining categories (e.g., for 4 + 1, the “other” data file contained a random selection of categories except for I20, I21, I25, and I50). To achieve equal representation, training data of the smaller categories were augmented up to four times. Table 5 shows the numbers of training and testing examples per class for each of the major training subsets.

We set up the fine-tuning process using the AdamW optimizer and a learning rate of 5 × 10^−7^. For Polish RoBERTa, we chose a batch size of 34, the maximum allowed by the memory of the NVIDIA A100 unit, and allowed the setup to run for up to 20 epochs. After each training run, we selected the final model from all epoch checkpoints by looking for the best performance on the validation set.

**Figure 3:**
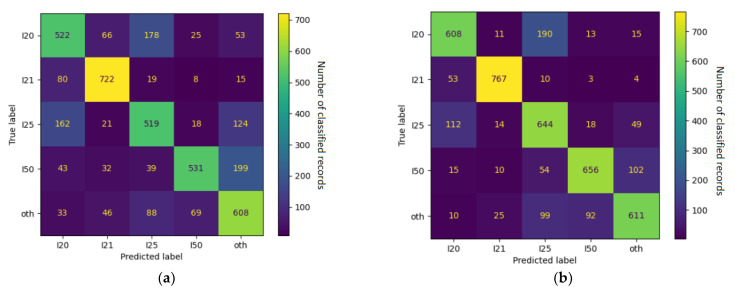
Confusion matrices for the 4 + 1 models trained on (**a**) admission data and (**b**) discharge data. Numbers refer to counts of examples in the respective categories, and the color spectrum reflects the contrast in their proportions.

**Figure 4:**
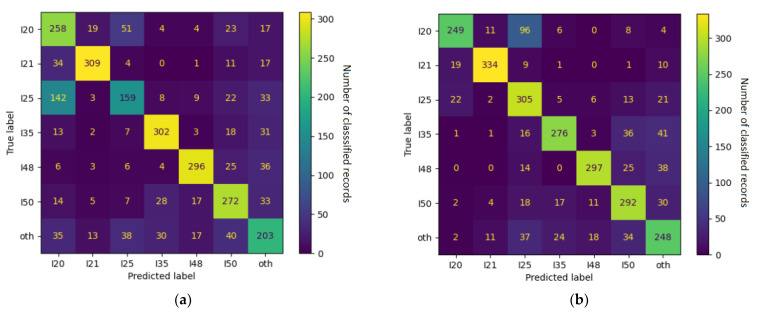
Confusion matrices for the 6 + 1 models trained on (**a**) admission data and (**b**) discharge data. Numbers refer to counts of examples in the respective categories, and the color spectrum reflects the contrast in their proportions.

For each training subset, we separately fine-tuned models with admission data (Section 1) and discharge data (Section 3). In the 4 + 1 and 6 + 1 training subsets, the accuracy (evaluated on a test set with balanced numbers of categories) approached 70% with admission data and 80% with discharge data (detailed results are shown in Table 5, and confusion matrices are shown in Figure 3 and Figure 4).

The 9 + 1 and 12 + 1 subsets gravitated toward accuracies of 60% and 70% for admission and discharge data, respectively, while staying well above baselines.

**Figure 5:**
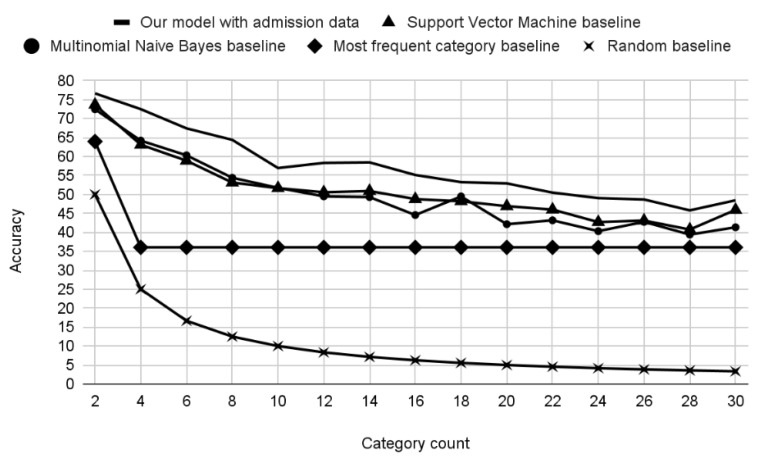
Chart showing the falloff of accuracy as the number of categories increases. The most frequent category baseline was calculated based on category frequencies in the entire corpus.

To visualize the role of category count in the models’ performance, we ran a set of fine-tuning experiments, gradually increasing the category count from 2 to 30, and observed a decline in accuracy (see Figure 5) that nonetheless stayed high compared to baselines such as random guess (48% vs. 3% for 30 categories) and the most frequent category (36%).

Note that the more categories are involved, the less data are available for the least populated ones, causing the further deterioration of accuracy and overfitting. With the presently available data, it would not be reasonable to increase the category count beyond 12 + 1 (all above 500 examples) or, at best, 15 + 1 (all above 300).

**Figure 6:**
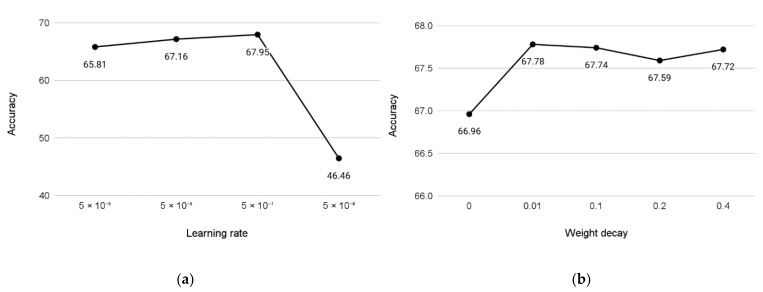
Sensitivity charts showing the changes in accuracy in relation to learning rate (**a**) and weight decay (**b**); training was conducted with the 6 + 1 category dataset version, limited to a maximum of 10 epochs.

The deep learning models need to set up several hyperparameters such as learning rate or weight decay, which are usually experimentally determined using a grid search. With our dataset, the optimization of learning rate and weight decay, as shown in Figure 6, indicated optimal values of 5 × 10^−7^ for learning rate and 0.01 for weight decay.

**Figure 7:**
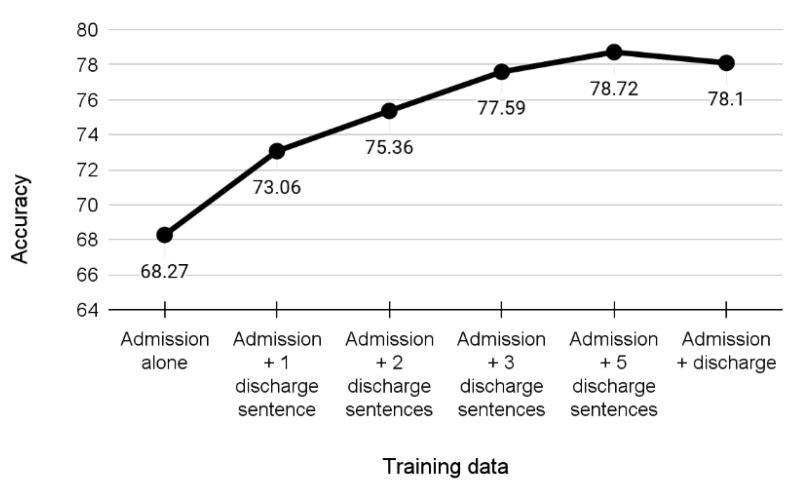
Rise in accuracy as admission training data are gradually extended with sentences from discharge text.

To further elucidate the relationship between admission and discharge data, training subsets that included the full admission text and a designated number of sentences from discharge text for each health record were created; see Figure 7. As the discharge text lists the procedures the patient has undergone, this experimental scenario “simulated” the process of gradually complementing the admission information with results of further investigations. The fact that adding just one sentence increased the accuracy by 4% with sustainable further improvements is a promising indicator.

Overall, the results show that a deep text analysis of very brief input summaries prepared at the time of patient’s admission to the hospital can provide an informed prediction of the final diagnosis. In case of distinguishing among the four most frequent diagnoses (plus a special “other” category), the model’s accuracy reached more than 68%. When increasing the number of diagnoses up to 30 (covering more than 98% of patients), the accuracy still stayed above or around 50%, i.e., more than 10 times higher than a random guess. Another implication of the results is that prediction based on the admission summary can be further improved (up to 78%) using from one to five sentences from the discharge summary that can be used to represent brief descriptions of investigations taking place during hospitalization.

## 4. Discussion

### 4.1. Error Analysis

As already indicated, the imperfect classification performance achieved with deep learning is mainly due to the constraints of the text modality in medicine—the data lack the multimodal and multi-format information involved in treating and diagnosing actual human beings.

Underlying this, the more fine-grained failures seem to be related to the specificity of the respective ICD-10 categories.

While I21 (acute myocardial infarction) can be expected to be easily distinguishable because of its acute nature, I48 (atrial fibrillation and flutter) presents with a very specific measurable symptom, and I35 (nonrheumatic aortic valve disorders) is explicitly localized in the aortic valve, other categories often lack this degree of specificity.

I25 (chronic IHD), by far the most populous ICD-10 category in our dataset, trailed behind other categories in terms of performance. This could have partially been due to the breadth of the category, which subsumes an unusual number of codes and conditions, but also due to its chronicity—patients suffering from chronic heart disease can be expected to be hospitalized in a variety of settings due to a variety of reasons, and the outcomes of treatment might range from finding very little to various isolated manifestations and interventions, diluting the specificity of the category for classification systems.

Similarly, I10 (essential hypertension) shares its chronic nature, various possible reasons for hospitalization, and inconclusive discharge statements with I25.

The second and fourth most frequent ICD-10 categories in our dataset, I20 (unstable angina pectoris) and I50 (heart failure)—neither as chronic nor bad-performing as I25 and I10—serve to highlight the principle that broad categories with low specificity tend to present below-average performance in classification.

Table 6 also shows that the worst performing category of all was found to be “other”, which is a topic in its own right. This category is necessary in order to enable the classifier to issue true statements about the data. However, the designation means “any of the rest” of the total of 170 ICD-10 categories that, being rarer, are often highly specific. Plus, these smaller categories, more often than resembling each other, are marginally related to one of the larger categories. Thus, this inverted set of highly diverse training examples is difficult to learn by its very design.

**Figure 8:**
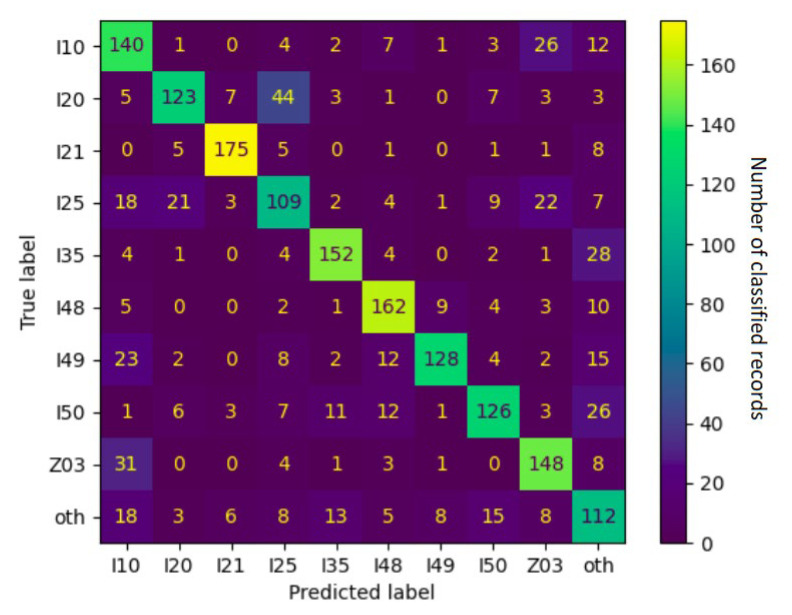
Confusion matrix of a 9 + 1 setup fine-tuned on both admission and discharge data. Numbers refer to counts of examples in the respective categories, and the color spectrum reflects the contrast in their proportions.

Upon the closer inspection of the confusion matrix in Figure 8, we can see that the notoriously underperforming I25 (chronic IHD) was often misclassified as I10 (essential hypertension), I20 (unstable angina pectoris), and Z03 (suspected condition not found). These relationships were often mutual—I20 tended to be misclassified as I25 even more often and I10 was classified as Z03 just as often as the other way around. Together with the fact that they all belong to the vaguer end of the spectrum, this indicates objective reasons for the classifier’s underperformance—i.e., actual overlap in the reported symptoms and physical circumstances between the ICD-10 categories.

Some of the relations were found to be unilateral: I25 tended to be misclassified as Z03, but Z03 was rarely ever thought to be I25; I25 was often regarded as I10, but I10 was unlikely to be classified as I25. This is even more telling regarding the relationships between ICD-10 categories because it specifies exactly where the unclarity resides. In this example, border cases of I25 frequently resembled the less serious categories of I10 and Z03 but not vice versa. The real-world correlate of this might be the preventive caution exercised in cardiology, calling attention to anomalies (however small) so that no potentially dangerous condition is left unattended.

The capability of classifiers to discover such relationships, especially if applied to larger datasets, has the potential to feed back into the medical domain and help reflect on the practices of differential diagnosing, possibly even drawing attention to previously unnoticed connections.

### 4.2. Medical Implications

The amount of data collected on a daily basis from hospital and outpatient healthcare systems is continuously growing [25]. In order to organize gathered medical information, the ICD classification system was adopted by healthcare institutions worldwide to assign diagnosis codes into EHR summarizing patients’ medical encounters.

In light of public health challenges, CVDs remain the leading cause of death globally [1,26,27]. Among them, CADs cover a group of clinical syndromes characterized by an imbalance between myocardial blood supply and demand that results in myocardial ischemia due to atherosclerotic plaque in the coronary arteries [28]. A broad spectrum of CAD includes chronic coronary syndromes [29,30] (also referred as chronic IHD or stable CAD [I25]) and acute coronary syndromes covering unstable angina pectoris (I20) and myocardial infarction (I21). As a consequence, CADs may lead to ischemic cardiomyopathy, defined as HF (I50), which is diagnosed in 1–2% of the adult population [31,32,33,34,35]. Furthermore, atrial fibrillation, one of the most prevalent cardiac arrhythmias, is a common comorbidity in HF patients, and both diseases have seen a rising number of incidences in recent years [36,37]. This has subsequently translated into a high number of outpatient and inpatient visits, generating tremendous amounts of medical information collected during routine clinical care. Medical providers process and organize these data into contextual information documenting them in the EHR system as clinical notes. However, the majority of information in the electronic documentation is stored in an unstructured format, making it challenging to analyze at scale [38,39]. Interestingly, advancements in the field of AI and NLP have enabled the in-depth evaluation of electronic medical data for research purposes, which, in turn, has strong practical potential.

For example, AI/NLP-based systems can be used to verify potential discrepancies between EHR-derived original ICD codes manually entered by clinicians and automatically generated ICD codes. Inappropriate diagnostic codes are being reported in an increasing number of publications including cases of stroke [40], myocardial infarction [41], and endocarditis [42]. Tremendous discrepancies were also reported in the ambulatory care in documenting ICD-10 codes for six standardized clinical case scenarios. Only half of provided codes were appropriately annotated by clinicians, while approximately a quarter of ICD-10 codes were missing [43]. Furthermore, a study on barriers affecting coding quality reported variability in the documents used for coding, increases in errors during transcriptions from paper due to extra actors, difficulties in choosing an appropriate code, and coding delay due to lack of resources and tools for coders [44].

From a clinical perspective, miscoding may have serious negative consequences for patients. On the other hand, the analyses of high quality EHR-derived data may provide predictive models showing clinical trajectories for specific patient cohorts, as well as phenotype subsets of diseases [41,45,46,47,48]. In a broader perspective, beyond automatic coding, the NLP-based approach has allowed for the building of predictive models [49] and the phenomapping analyses of individuals with myocardial infarction [50] and heart failure, which reflects heterogeneous clinical syndrome [51,52,53]. Novel classification may help to define specific therapeutic strategies in this challenging group of HF patients [54]. Moreover, multi-modal algorithms searching for myocardial infarction-related keywords, ICD codes, and information on percutaneous coronary intervention procedures in discharge summaries have increased the positive predictive value of detecting the ST-segment elevation myocardial infarction type in EHRs [55].

Importantly, erroneous coding impacts hospital-level quality metrics, having a broader influence on epidemiological studies that are used to build public health strategies [56]. For example, the SILesian CARDiovascular (SILCARD) registry, built in collaboration between the Silesian Centre for Heart Diseases in Zabrze and the Regional Department of the Polish National Health Fund, was used to analyze causes of hospitalization and prognosis in CVD patients of the entire Silesian region inhabited by 4.6 million people. Specifically, data from 310 hospital departments and 1863 outpatient clinics specialized in cardiology, cardiac surgery, diabetology and vascular surgery contained information on 487,518 patients and 956,634 inpatient encounters. The primary ICD-10 and ICD-9 codes were used for statistical analysis, reporting high prevalences of HF and CADs, as well as declining trend in 1-year mortality among CVD patients [57]. Similarly, populational trend evaluations were performed for atrial fibrillation [58], left atrial appendage occlusion procedures [59], transcatheter aortic valve implantation and surgical aortic valve replacement operations [60], implantable cardioverter-defibrillators and cardiac resynchronization therapy [61]. Furthermore, NLP technology allows for the in-depth EHR assessment of social determinants, which are non-medical factors impacting patient health outcomes [62,63,64]. Leveraging this opportunity, AI systems can help to verify the correctness of the diagnoses, as well as provide valuable information on critical aspects associated with populational health.

It should be also mentioned that misclassification and inaccuracy in diagnostic codes are directly associated with the reimbursement process for healthcare institutions. For example, the down-grading of the ICD-10 code (i.e., miscoding I25 instead of I21) will categorize a CAD as chronic IHD instead of acute myocardial infarction, which has a higher billing rate. While discussing the economic perspective, it should be mentioned that the adoption costs of the novel ICD system are substantial and included the training of the users, as well as initial and long-term losses of productivity. In the U.S., it was estimated that costs of ICD-10 implementation ranged from $425 million to $1.15 billion, adding $5–40 million per year in lost productivity [65].

Another important aspect might be exemplified by the opportunity to reduce the documentation-related burden imposed on medical staff. Data from a cardiology outpatient clinic show medical providers spend approximately 50% of their time with electronic documentation and only 30% with patients [4]. Of note, the ICD-10 coding process is time- and resource-consuming due to the complexity of coding rules (e.g., code orders, inclusion/exclusion criteria, and growing number of ICD-10 codes). It was estimated that for a professional disease coder, ICD-10 categorization may take ~0.5 h per case. Thus, an automatic AI-based system for imputing ICD-10 codes from free-text format might be implemented and synchronized with existing EHR systems to detect, red-flag, and potentially correct misclassified diagnostic codes. This could pave a way to ensure reliable clinical, administrative and reimbursement data for everyday practical applications and for research-oriented advanced downstream analysis [3,66].

For this purpose, we explored the electronic medical database of GCM hospital, which is one of the largest hospitals in Poland. The Cardiology and Cardiac Surgery Centre of GCM has been specializing in the most complex medical procedures for over 35 years. Specifically, we explored the 3rd Department of Cardiology, which consists of 52 beds, including 12 cardiac intensive care unit beds, that generate a vast amount of non-English language medical data available for analysis. In light of the limited availability of standard medical vocabularies and NLP tools for Polish language information extraction, we aimed to test the efficacy of the current best deep learning models when predicting a patient’s diagnoses based on small selected subsets of the patient’s medical history. Our results demonstrated that the evaluated models significantly surpass other techniques and can offer fast and well-timed estimates of necessary follow-up procedures. From a practical standpoint, we plan to use the results of the current study to perform phenomapping analyses of patients presenting to the emergency department with chest pain to improve differential diagnosis efficacy. As a foundation, this approach could support medical providers in everyday clinical practice. Furthermore, we aim to apply the NLP/AI framework to simulate an economic impact of potentially miscoded diseases on the reimbursement process. It is essential for healthcare institutions to consider the financial aspect which is crucial to secure quality medical supplies, provide access to novel technologies and offer high standards of care.

## 5. Conclusions

Automated ICD coding systems have the potential to support providers in everyday clinical practice, help medical organizations to optimize administrative/reimbursement processes, and reduce costs for healthcare systems during the implementation of novel coding systems.

We have introduced a new deep learning model for processing summary texts of Polish electronic health records for the task of predicting final patient diagnoses. The presented text analysis model was shown to be able to predict the diagnosis code of the four prevailing diagnoses that cover 75% of cases with 69–78% accuracy based on the mix of its input texts. The 69% accuracy can be achieved immediately after the patient admission to the hospital using just the medical history text (132 words in average), and the accuracy can be increased to 78% by adding further examination results (represented by 1–5 successive single sentences from the discharge summary). As the model can be applied as an early prediction support, its current form can already offer valuable data to doctors and to medical administration.

In future work, we plan to improve the accuracy of the model by constructing an ensemble of fine-grained diagnosis predictors that concentrate on a specific subset of diagnoses and can thus concentrate on the discriminating details. We will also provide prediction feedback to the medical specialists regarding the most frequent misclassification to discuss potential regular inconsistencies in their summaries.

## Figures and Tables

**Table 1:** Statistics detailing unit counts and averages in the Polish health record dataset.

Average word count per record	472
Average word counts per sections	132; 249; 86; 64
Records	50,465
Sentences	2,583,087
Words	23,831,785
Tokens	34,315,153

**Table 2:** Example of a complete health record in the Polish health record dataset.

Section 1 (Admission)	Pacjent przyjęty w ramach ostrego dyżuru z powodu zawału mięśnia sercowego ściany dolnej. Spoczynkowe dolegliwości wieńcowe od 29.03. 17:00, w dniu dzisiejszym, w godzinach porannych zgłasił sie do poradni, gdzie rozpoznano zawał. Czynniki ryzyka choroby wieńcowej: Hypercholesterolemia i nadcisnienie leczone skutecznie. Nigdy nie palił. Cukrzycę neguje, ale przy przyjęciu cukier >200 mg% i dodatni wywiad rodzinny—matka chorowała. Skargi dodatkowe i choroby przebyte: Nie zgłasza. Na żółtaczkę nei chorował, nie szczepiony. Alergie i nietolerancje lekowe neguje. Wywiad rodzinny: Matka chorowała na cukrzycę. Bez wczesncyh powikłań miażdżycowych w rodzinie.
Section 2 (Physical)	Pacjent przytomny, ułożenie dowolne, kontakt logiczny zachowany. Budowa prawidłowa, nadwaga 170 cm, 90 kg. Skóra prawidłowo ucieplona, bez wykwitów patologicznych. Tkanka podskórna prawidłowo rozwinięta. Węzły chłonne niewyczuwalne. Głowa opukowo niebolesna. Gałki oczne osadzone prawidłowo, symetryczne. Źrenice równe, okrągłe, prawidłowo reagują na światło i nastawność. Nad płucami wypuk jawny, szmer oddechowy pęcherzykowy symetryczny. Drżenie głosowe zachowane. Akcja serca miarowa 80/min. Tony serca głuche, bez szmerów patologicznych. Brzuch miękki, palpacyjnie niebolesny, bez oporów patologicznych. Wątroba pod łukiem żebrowym. Śledziona, nerki niewyczuwalne. Objawy Chełmońskiego i Blumberga ujemne. Objaw Goldflama obustronnie ujemny. Perystaltyka słyszalna. Bez obrzęków obwodowych. Bez zmian żylakowatych. Tętno na tt. kończyn dolnych dobrze wyczuwalne.
Section 3 (Discharge)	Pacjent lat 68 przyjęty w ramach ostrego dyżuru z powodu zawału ściany dolnej mięśnia sercowego. Wykonano koronarografię, w której stwierdzono w prawej tętnicy wieńcowej w początkowym odcinku 99% zwężenie. Jednoczasowo wykonano skuteczny zabieg PCI PTW z implantacją stentu. W lewej tętnicy wieńcowej stwierdzono: pień bez zmian, LAD bez zmian, LCx zmiany przyścienne, OM1 dość szeroka i rozległa, medialnie krótka zmiana do 95%. Wskazany w 2-gi etap PCI w OM1. Przeprowadzono wewnatrzszpitalny etap rehabilitacji kardiologicznej. W badanich dodatkowych stwierdzono podwyższone wartości glikemii, rozpoczęto intensywną farmakoterapię (z insuliną). Pacjent wypisany do domu z zaleceniami jw.
Section 4 (Recommendations)	Vivacor 6,25 1-0-1 Enarenal 5 1-0-1 Polocard 75 mg 0-0-1 Zocor 20 mg 0-0-1 Ranigast 150 mg 0-0-1 Plavix 1-0-0 (optymalnie 12 miesięcy) Siofor 500 mg 1-1-0 Insulina wg. poziomu glukozy (ostatnie zapotrzebowanie: NovoMix 30: R-20j, W-19j) Normalizacja wagi ciała. Dieta cukrzycowa.Dalsze leczenie w Poradniach: lekarza rodzinnego, kardiologicznej, diabetologicznej (pilne). Pacjent za około 2 miesiące zostanie ponownie przyjęty celem wykonania 2-go etapu leczenia choroby wieńcowej (PCI OM)- konieczne skierowanie do Kliniki. Po zakończeniu leczenia interwencyjnego proponujemy rehabilitacje w warunkach sanatoryjnych.
ICD-10 diagnosis	I21.1

**Table 3:** Overview of the 10 most frequent concluding primary ICD-10 categories in the dataset. This distribution does not reflect realistic diagnosis frequencies because the conditions frequently co-occur and one health record in the dataset can only be assigned one diagnosis code.

ICD-10 Category	Medical Name	Count in Dataset
I25	Chronic ischemic heart disease/chronic coronary syndrome	17,973 (36.03%)
I20	Unstable angina pectoris	9741 (19.53%)
I21	Acute myocardial infarction	6262 (12.55%)
I50	Heart failure	3360 (6.74%)
I48	Atrial fibrillation and flutter	1678 (3.36%)
I35	Nonrheumatic aortic valve disorders	1511 (3.03%)
I10	Essential hypertension	1299 (2.60%)
I49	Other cardiac arrhythmias	881 (1.77%)
Z03	Suspected condition not found	804 (1.61%)
I34	Nonrheumatic mitral valve disorders	777 (1.56%)

**Table 4:** Overview of the training subsets listing individual ICD-10 categories included in the training and coverage of such a subset with respect to the whole dataset.

Training Subset	Categories Included	Coverage
4 + 1	I25, I20, I21, I50, “other”	74.8%
6 + 1	I25, I20, I21, I50, I48, I35, “other”	81.2%
9 + 1	I25, I20, I21, I50, I48, I35, I10, I49, Z03, “other”	87.2%
12 + 1	I25, I20, I21, I50, I48, I35, I10, I49, Z03, I34, Q21, I42, “other”	91.0%

**Table 5:** Overview of the main classification results for the different dataset variations.

Training Dataset	Training/Testing Examples per Class	Accuracy (Admission Data)	Accuracy (Discharge Data)
4 + 1	15,148/841	68.79%	78.64%
6 + 1	6773/376	67.71%	77.00%
9 + 1	3533/196	59.62%	71.49%
12 + 1	2458/136	56.49%	69.21%

**Table 6:** Performance per category in a 9 + 1 setup fine-tuned on both admission and discharge data, sorted by F1 score, as a harmonic mean of precision and recall.

ICD-10 Category	F1 Score	Frequently Confused with
I21	0.90	other
I48	0.80	other, I49
I35	0.79	other
I49	0.74	I10, other, I48
Z03	0.72	I10
I20	0.69	I25
I50	0.69	other, I48, I35
I10	0.63	Z03, other
I25	0.56	Z03, I20, I10
other	0.53	I10, I50, I35

## Data Availability

Non applicable.
